# Supplementary material for: A Serosurvey of Japanese Encephalitis Virus in Monkeys and Humans Living in Proximity in Thailand
Source: Viruses. 2023 May 9;15(5):1125. doi: 10.3390/v15051125 (PMC10221860; doi:10.3390/v15051125)
Supplement: Supplementary file 1 [file viruses-15-01125-s001.zip › viruses-2292664-supplementary.pdf]

Table S1. Seropositivity of neutralizing JEV antibody in monkey samples.

	Study site 1	Study site 2
No. seropositive[1]	15/102 (14.7%)	6/108 (5.6%)

[1] Data presented as n positive/n total (% of total)

Table S2. Seropositivity of neutralizing JEV antibody in human samples.

	Study site 1	Study site 2
No. seropositive[1]	55/126 (43.7%)	52/115 (45.2%)

[1] Data presented as n positive/n total (% of total)
